# Common Spontaneous Tumors and Tumor-like Lesions in 70 Pet Rodents and Negative MMTV Detection in Mammary Tumors

**DOI:** 10.3390/ani14101469

**Published:** 2024-05-15

**Authors:** Ya-Mei Chen, Jia-Ling Wu, Wei-Hao Lin

**Affiliations:** College of Veterinary Medicine, National Pingtung University of Science and Technology, Neipu, Pingtung County 912301, Taiwanwhlin@mail.npust.edu.tw (W.-H.L.)

**Keywords:** pet rodents, spontaneous tumors, MMTV, hamsters, guinea pigs, mammary gland tumors

## Abstract

**Simple Summary:**

Rodents have become increasingly popular as household pets, but few studies have focused on tumors in pet rodents. Pet rodents differ from their laboratory counterparts in many aspects such as geographical preference of pet species or breeds, household raising conditions, population density, and life expectancy. Therefore, it is worth conducting this pathological study of 77 spontaneous tumors from 70 pet rodents. Overall, mammary gland tumors were common. In the meantime, mouse mammary tumor virus (MMTV)-like sequences were found in the tissues of dogs, cats, and humans, raising the concern of whether these pets may incidentally serve as a vehicle for the cross-species transmission of MMTV to household personnel. For that purpose, nuclei acid extracted from the formalin-fixed paraffine-embedded tissues of 20 rodent mammary gland tumors was detected for MMTV *env* gene sequences. All tumors revealed negative results of MMTV detection.

**Abstract:**

Compared to the number of studies on the neoplasms of laboratory rodents, fewer studies have focused on spontaneous neoplasms in pet rodents. Notably, the mouse mammary tumor virus (MMTV) is associated with mammary tumors in rodents. In this study, 77 tumors and tumor-like lesions of biopsy samples were collected from 70 pet rodents, including hamsters (n = 47), guinea pigs (n = 16), unknown species (n = 4), rats (n = 2), and a gerbil. Fifty tumors were collected from 47 hamsters, in which the most common tumors were mammary tumors (13/50), followed by fibrosarcoma (9/50), mast cell tumors (4/50), and squamous cell carcinoma (4/50). The collected subtypes of mammary tumors in hamsters included tubular carcinoma (n = 5), tubular adenoma (n = 4), carcinoma and malignant myoepithelioma (n = 1), simple tubular carcinoma (n = 1), adenosquamous carcinoma (n = 1), and tubulopapillary adenoma (n = 1). In addition, twenty tumors were collected from guinea pigs, in which the most common tumor was lipoma (6/20), followed by adenocarcinoma of the mammary gland (4/20), trichofolliculoma (2/20), and collagenous hamartomas (2/20). In guinea pigs, the subtypes of mammary gland tumors were tubular carcinoma (n = 2), tubular and solid carcinoma (n = 1), and tubulopapillary carcinoma (n = 1). In 20 cases of mammary tumors, MMTV was not detected, implicating no evidence of MMTV infection in mammary oncogenesis in pet rodents in Taiwan.

## 1. Introduction

Examples of rodents that are kept as household pets include mice, rats, guinea pigs, hamsters, gerbils, and chinchillas. Among these rodents, hamsters and guinea pigs are welcomed pets in Taiwan. These hamsters included Syrian hamsters or golden hamsters (*Mesocricetus auratus*) and dwarf hamsters, which comprise *Phodopus sungorus*, *Phodopus campbelli*, *Phodopus roborovski*, and *Cricetulus griseus*. The average life expectancy of hamsters is 24 to 36 months [1]. Guinea pigs (*Cavia porcellus*) belong to the genus *Cavia* of the family *Caviidae*, with an average life expectancy of 4 to 5 years in the wild and up to 8 years for pets [2].

In contrast to the well-controlled environment for laboratory rodents, the environment where pet rodents live is highly variable. Without the restricted management of biological controls, it is presumed that pet rodents may have a greater chance of being exposed to pathogens, resulting in more infectious as well as noninfectious diseases. Due to the complexity of the gene background and environment, these pet rodents are no longer considered proper animal models. Spontaneous neoplasms are an important disease in senile pets. Three studies have described the tumors of pet hamsters in Japan, Germany, and Brazil [3,4,5]. Compared to Syrian hamsters, dwarf hamsters have a greater incidence of tumors. The cause of the higher incidence is unrevealed [3]. The most commonly affected system is the integumentary, followed by the hematologic or the female reproductive systems. In guinea pigs, the most common tumor is trichofolliculoma.

Mouse mammary tumor virus (MMTV) belongs to the genus *Betaretrovirus* of the *Retroviridae* family. MMTV binds to transferrin receptor 1 (TfR1) on host cells via the Env protein [6]. In host cells, viruses synthesize double strands of DNA that connect to the chromatin of host cells, leading to tumor transformation. Mice receive MMTV via both endogenous and exogenous routes. In the endogenous route, infected eggs or sperm pass the virus to offspring. In the exogenous route, mice receive viruses from milk through dendritic cells and lymphocytes in the gastrointestinal system. In addition to mice, other rodents, including buffalo rats and hamsters, are infected with MMTV [7,8,9]. Moreover, MMTV *env* gene-like sequences are found in human breast cancers, implying a potential zoonotic risk between humans and pet rodents [10,11].

This study aimed to retrospectively collect the tumors and tumor-like lesions from biopsy samples of pet rodents in Taiwan. This study also investigated the role of MMTV in mammary gland tumors from pet rodents.

## 2. Materials and Methods

### 2.1. Case Collection

This study retrospectively collected biopsy samples from pet rodents. All specimens in this study were submitted from animal hospitals to the Animal Disease Diagnostic Center (ADDC), College of Veterinary Medicine, National Pingtung University of Science and Technology (NPUST) in Taiwan. The collection period spanned from 2012 to May 2023, and collected information included species, age, sex, neuter status, and tumor location and size. The total number of biopsy samples was 77.

### 2.2. Histopathology

Tissues were fixed in 10% neutral buffered formalin for no more than 48 h. Fixed tissues were routinely processed and embedded in paraffin, and 4 μm-thick sections were stained with hematoxylin and eosin (HE). Diagnosis was confirmed by two veterinary pathologists.

The classification of mammary tumors in this study was based on that of canine mammary tumors [12], wherein benign tumors include simple adenomas, intraductal papillary adenomas, ductal adenomas, fibroadenomas, myoepitheliomas, complex adenomas, and benign mixed tumors. On the other hand, malignant tumors include carcinoma—in situ, carcinoma—micropapillary invasive, carcinoma—solid, comedocarcinoma, carcinoma—anaplastic, carcinoma—complex-type, carcinoma and malignant myoepithelioma, carcinoma—mixed type, ductal carcinoma, and intraductal papillary carcinoma.

### 2.3. Nested Polymerase Chain Reaction (Nested PCR)

Eight serial 10 μm-thick formalin-fixed paraffin-embedded (FFPE) tissue sections were collected from each tumor. Then, the DNA was purified via a QIAamp^®^ DNA FFPE Tissue Kit (cat. 56404; Qiagen, Germantown, MD, USA) and stored at −20 °C.

The primers used for nested PCR to detect MMTV originated from Wang’s study [11,13]. The primers used targeted the MMTV *env* gene with expected product sizes of 647 and 449 bp (Table 1). The reaction mixture consisted of ultrapure distilled water (DNase and RNase free), 10 μL of Phire Green Hot Start II PCR Master Mix (Thermo Scientific^TM^, Waltham, MA, USA), and 5 μM primers. The sample tubes contained 2 μL of DNA extracted from the tumor specimens. The negative controls consisted of the reaction mixture alone, while the positive control contained 1 μL of viral genomic DNA from MMTV-infected NIH 3T3 cells, which were kindly provided by Prof. Wei-Li Hsu from the National Chung Hsing University.

For the nested PCR, the following protocol was used with 1 μL of the amplicon from the first PCR used as the template for the second amplification: 1 cycle at 98 °C for 30 s and 35 cycles at 98 °C for 5 s, 63 °C for 5 s, and 72 °C for 1 min, followed by a final extension cycle at 72 °C for 1 min. PCR amplicons and a DNA marker (ExcelBand™ 100 bp+3K DNA Ladder, Smobio^®^, Hsinchu city, Taiwan) were then electrophoresed in a 2% agarose gel in 1× TAE buffer at 100 V for 32 min. The gels were then stained with Healthview^TM^ nucleic acid stain (Genomics^®^, New Taipei City, Taiwan) and imaged on a gel-doc imager by using multianalyst software Version 1.1 (Bio-Rad, Hercules, CA, USA).

## 3. Results

From 2012 to May 2023, the ADDC of NPUST received 77 tumor-like masses from 70 pet rodents. The collected species included hamsters (n = 47), guinea pigs (n = 16), unknown species (n = 4), rats (n = 2), and a gerbil (n = 1).

### 3.1. Hamsters

In the hamster cases (n = 47), 9 were from Syrian hamsters, 10 were from dwarf hamsters, and 28 were from unspecified breeds. There were 22 cases from males and 25 cases from females, and 90.9% of the tumors from males (20/22) and 92% of the tumors from females (23/25) were intact. The median age was 18 months.

**Figure 1:**
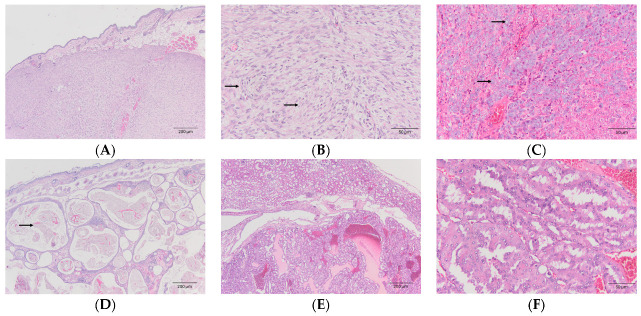
Tumor examples from hamsters. Hematoxylin and eosin (HE) staining. (**A**,**B**) Fibrosarcoma (40× and 200×, respectively). The neoplastic cells were arranged in stream pattern (arrows in (**B**)). (**C**) Mast cell tumor (200×). Neoplastic cells had abundant amount of grey cytoplasmic granules (arrows). (**D**) Pilomatricoma (40×). The neoplasm forms multiple cystic structures containing keratin and ghost cells (arrow). (**E**,**F**) Renal cell carcinoma (40× and 200×, respectively). Neoplastic cells were arranged in tubular pattern and compressed the adjacent parenchyma.

A total of 50 masses were diagnosed and the most commonly affected system was the integumentary (41/50), followed by the reproductive system (4/50) and the urinary system (3/50). Overall, the most common diagnosis was malignant mammary tumors (10/50), all from females, followed by fibrosarcoma (9/50), mast cell tumors (4/50), and SCC (4/50) (Table 2). Besides the histological features, the diagnosis of mast cell tumors was also confirmed by T blue staining. Within the integumentary system, the most common tumors were malignant tumors of the mammary gland (10/41), fibrosarcoma (9/41) (Figure 1A,B), mast cell tumors (4/41) (Figure 1C), and SCC (4/41). Regarding the hair follicle origin, there was one case of pilomatricoma (Figure 1D) and trichoepithelioma, respectively. In the female reproductive system, the diagnoses included luteoma (n = 1), a granulosa cell tumor (n = 1), and uterine leiomyosarcoma (n = 1). In the male reproductive system, a seminoma was diagnosed. In the urinary system, all individuals were diagnosed with renal cell carcinoma (n = 3) (Figure 1E,F).

### 3.2. Guinea Pigs

**Figure 2:**
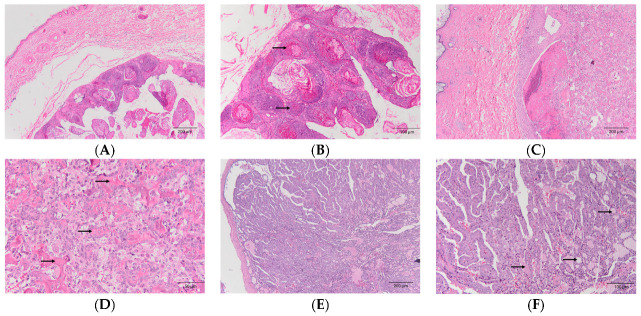
Tumor examples from guinea pigs. Hematoxylin and eosin (HE) staining. (**A**,**B**) Trichofolliculoma (40× and 100×, respectively). The neoplasm consisted of multiple follicles in various stages (arrows in (**B**)). (**C**,**D**) Osteosarcoma (40× and 200×, respectively). The neoplasm invades adjacent subcutis and forms multiple osteoid (arrows in (**D**)). (**E**,**F**) Thyroid adenocarcinoma (40× and 100×, respectively). Neoplastic cells were arranged in tubular pattern and contained variable amounts of thyroid colloid (arrows in (**F**)).

A total of 20 tumors were collected from 16 guinea pigs in this study. There were eight cases from males and eight cases from females, and 75% of the males (6/8) and 100% of the females (8/8) were intact. The median age was 21 months. Overall, the most common tumor was lipoma (6/20), followed by an adenocarcinoma of the mammary gland (4/20), and collagenous hamartoma (2/20) (Table 3). It is interesting to note that more mammary tumors came from males (n = 3) than from females (n = 1). The adjacent normal mammary gland around the tumor indicates the origin of the mammary gland instead of the apocrine gland. The most commonly affected system was the integumentary (15/20), followed by the reproductive system (2/20) and skeletal system (2/20). In the integumentary system, the most common tumors were lipoma (5/15), an adenocarcinoma of the mammary gland (4/15), trichofolliculoma (2/15) (Figure 2A,B), and collagenous hamartoma (2/15). In the reproductive system, the diagnoses were uterine adenocarcinoma (1/15) and uterine lipoma (1/15). In the skeletal system, the diagnoses were osteosarcoma (1/15) (Figure 2C,D) and a malignant giant cell tumor of the bone (1/15). In the endocrine system, there was a case of thyroid carcinoma (Figure 2E,F).

### 3.3. Rats and Gerbil

Two intact female rats, aged 9 and 19 months, respectively, were collected in this study. The biopsy samples from the rats were diagnosed as mammary gland adenocarcinoma and fibroadenoma of the mammary gland, respectively. There was only one gerbil collected in this study. The animal was an intact 32-month-old male with a diagnosis of cutaneous melanoma of the left chest.

### 3.4. MMTV Detection in Rodent Mammary Tumors

**Figure 3:**
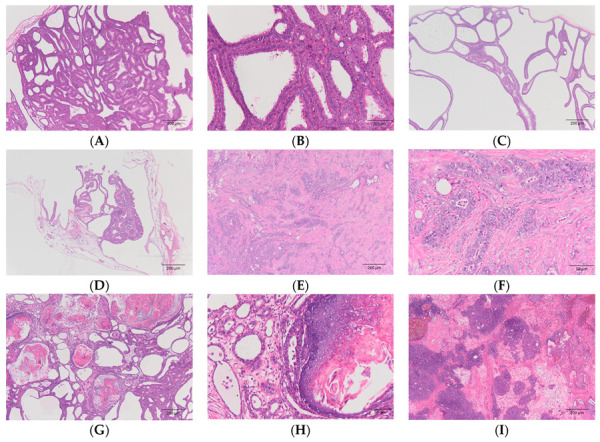

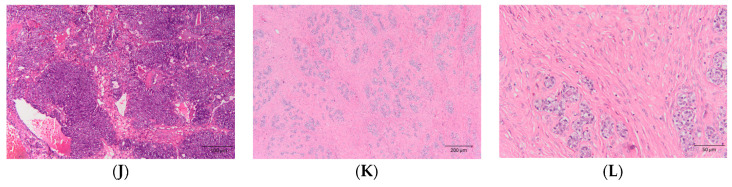
Tumors of mammary gland. Hematoxylin and eosin (HE) staining. (**A**,**B**) Tubular carcinoma from a hamster (40× and 200×, respectively). (**C**,**D**) Tubulopapillary adenoma from a hamster (40×). (**E**,**F**) Carcinoma and malignant myoepithelioma from a hamster (40× and 200×, respectively). (**G**,**H**) Adenosquamous carcinoma from a hamster (40× and 200×, respectively). (**I**,**J**) Tubular carcinoma from a guinea pig (40× and 100×, respectively). (**K**,**L**) Fibroadenoma of the mammary gland from a rat (40× and 200×, respectively).

**Figure 4:**
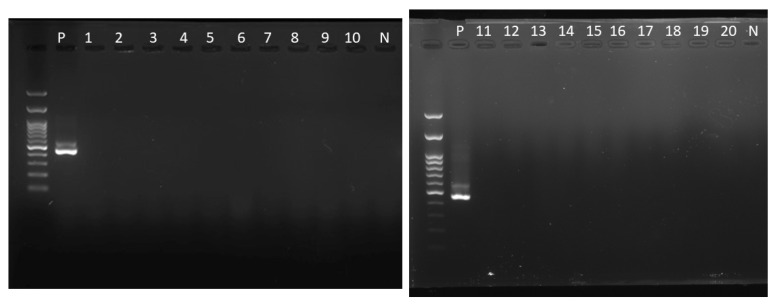
MMTV detection in mammary tumors in pet rodents. A total of 20 mammary tumors were detected as negative for MMTV using nested PCR targeted on *env* gene. The positive control was viral genomic DNA from MMTV-infected NIH 3T3 cells. P, positive control. N, negative control.

In this study, a total of 20 individuals were diagnosed with mammary tumors, collected from hamsters (n = 13), guinea pigs (n = 4), rats (n = 2), and unclear species (n = 1). The median age of affected hamsters was 14 months (range = 5–24), while the median age of affected guinea pigs was 55 months (range = 48–64). Among the hamsters, the subtypes of mammary tumors were tubular carcinoma (n = 7) (Figure 3A,B), tubulopapillary adenoma (n = 4) (Figure 3C,D), tubular adenoma (n = 1), carcinoma and malignant myoepithelioma (n = 1) (Figure 3E,F), and adenosquamous carcinoma (n = 1) (Figure 3G,H). In the guinea pigs, the subtypes of mammary gland tumors were tubular carcinoma (n = 2) (Figure 3I,J), tubular and solid carcinoma (n = 1), and tubulopapillary carcinoma (n = 1). Among the rats, the subtypes were mammary gland adenocarcinoma (n = 1) and fibroadenoma of the mammary gland (n = 1) (Figure 3K,L). All mammary tumors were subjected to MMTV detection, as described in Section 2.3, and all specimens were negative for the MMTV *env* gene (Figure 4), while the positive control was valid.

## 4. Discussion

This study retrospectively collected 77 spontaneous tumors and tumor-like lesions from 70 pet rodents in Taiwan spanning from 2012 to May 2023. The studied samples were mainly from hamsters and guinea pigs, which are the most popular pet rodent species in Taiwan. Information regarding why these pet rodents are popular in Taiwan is lacking since pet rodents are not required to register; therefore, the overall incidence of tumors cannot be estimated. We conjecture that hamsters are welcomed because of their solitary nature and small size, while guinea pigs are welcomed due to their docile personality. Surprisingly, no mouse samples were received in this study. We conjecture that the general public stereotypically regard mice and rats as animals for laboratory purposes rather than for pets. In the present study, the most commonly affected system was the integumentary system, followed by the reproductive system and urinary system. Presumably, the owners can easily notice and palpate these masses on the skin and thus increase their frequency of submissions for histopathology.

In the present study, 20 mammary gland tumors were collected from hamsters, guinea pigs, rats, and an unknown rodent. The median age of affected hamsters and guinea pigs were 14 and 55 months, respectively. Furthermore, all mammary gland tumors were collected from intact rodents. These findings suggest that, similar to other animals, mammary gland tumors occur in aged pet rodents and sex hormones might play a critical role in tumorigenesis [14]. Since mammary tumors are commonly observed in pet rodents, these tumors were further investigated for MMTV detection. To our knowledge, this is the first reported MMTV investigation in pet rodents. After the discovery of MMTV in mice, studies also revealed MMTV *env*-like sequences in humans, rhesus macaques, and cats [15]. In Taiwan, MMTV-like nucleotide sequences were detected in neoplastic and normal mammary tissue in both dogs and cats [16], in which the nucleotide sequences had 94–98% similarity with those in human breast cells. These findings raise the potential contribution of pets to the transmission of MMTV. It is suspected that cats received the virus from rodent prey while humans have direct contact with infectious secretions, such as saliva, from MMTV-infected animals [11]. Therefore, it is worth investigating the MMTV in pet rodents. Prompted by this concern and considering the daily interactions of pets with their owners, 20 rodent mammary tumors in this study (Figure 3) were tested for MMTV *env* gene sequences. Fortunately, the results turned out negative in these rodent mammary tumors, temporarily relieving the public of this concern. It is known that rats and dwarf hamsters harbor endogenous MMTV-like sequences [7,17]. For now, no research has focused on the observation of MMTV-like genes in guinea pigs. Nevertheless, an endogenous retrovirus can transmit to different host species when the virus is expressed at a high load [18]. Pet rodents can get a cross-species infection with pet or wild rodents in pet stores [19]. Together, these findings indicate that the transmission of MMTV between pet rodents and humans is doubtful and undefined.

A total of 47 hamsters were collected in the present study. The lifespan of hamsters is 18–36 months [1]. The median age of the affected animals was often over 12 months [3,4,5]; similarly, the median age of the hamsters in the present study was 18 months. The most common affected system in our collection was the integumentary, consistent with published findings [3,4,5]. According to a study of 177 pet hamsters in Germany, the most common skin tumor was the follicular tumor [3], while another study of 80 hamsters in Japan revealed that the most common tumor was a mammary gland tumor [5], similar with our findings in Taiwan (Figure 3). In the present study, there were more malignant mammary tumors than benign tumors and the subtypes included tubular adenoma, tubulopapillary adenoma, tubular carcinoma, carcinoma, malignant myoepithelioma, and adenosquamous carcinoma (Section 3.1). In a study of 45 cases of mammary tumors in domestic Siberian dwarf hamsters, the most common subtypes were adenocarcinoma, adenoma, and carcinosarcoma [20]. Both carcinoma and malignant myoepithelioma and carcinosarcoma have combined cellular populations, including glandular cells and spindle cells. Nevertheless, carcinosarcoma is characterized by balloon cells admixed with a polygonal cell population, which is not observed in our study. In both tumors, the spindle cells are immunopositive for p63 and smooth muscle actin (SMA) and negative for cytokeratin 8 (CK8) [20].

In the present study, the common skin neoplasms in hamsters also included fibrosarcoma, mast cell tumors, and SCC (Section 3.1). In southern Brazil, the most common neoplasm in 40 domestic hamsters was SCC, mostly located in the lip/nasal region [4]. According to Rother et al. (2021), papilloma and SCC in hamsters are commonly located in the head and extremities [3]. In the present study, three of the four cases involved the face. Together, these findings imply that the head is the preferred location for SCC in hamsters. In both the study from southern Brazil and the present study, the second most common skin tumor is fibrosarcoma [4]. Fibrosarcoma commonly occurs in the limb and abdomen [4]. In the present study, eight of the nine fibrosarcomas occurred in the abdomen. Meanwhile, mast cell tumors in hamsters are rarely reported in the literature [21]. Quite differently, another study suggested that the common integumentary tumors in domestic hamsters are scent gland tumors and cutaneous lymphoma [1]. Infections with hamster polyomavirus can lead to cutaneous lymphoma and trichoepithelioma [22]. In the present study, one case of trichoepithelioma and one case of round cell tumors were included. It is uncertain whether these cases are associated with viral infection.

The second most common system affected in hamsters in the present study is the female reproductive system, including luteoma, granulosa cell tumor, and uterine leiomyosarcoma (Table 2). According to a study in southern Brazil, leiomyosarcoma is the most common type of uterine tumor [4].

The third most common affected system in our study was the urinary system, and all three cases were renal cell carcinoma (RCC) (Table 2). Spontaneous RCC is rare but was reported in two Siberian dwarf hamsters (*Phodopus sungorus*) at Lincoln Park Zoo in Chicago in 1998 [23]. In the present study, the species of two cases were Syrian hamsters, and that of one case was an unknown breed. Male Syrian hamsters are an animal model of kidney tumors. These animals develop kidney tumors after treatment with estrogen, indicating a hormone-mediated process [24]. Interestingly, the Syrian hamsters in the present study were males, which is consistent with the sex difference. Nevertheless, whether estrogen plays a role in carcinogenesis in our cases remains unclear due to a lack of history.

The lifespan of guinea pigs is 5–7 years and spontaneous tumors usually occur at more than 3 years of age [25,26]. The median age in the present study was 21 months, ranging from 13 to 82 months, which was younger than the literature reported. In the present study, the most common tumor is cutaneous lipoma, followed by adenocarcinoma of the mammary gland and trichofolliculoma (Table 3). In a retrospective study of 493 animals, the most common tumors from externally palpable masses were lipoma, trichofolliculoma, lymphoma, mammary tumors, and thyroid carcinoma [26]. In another study involving 19 pet guinea pigs, the most common spontaneous tumors were tumors arising from hair follicles, mammary gland hyperplasia and tumors, and lipomas [27]. Trichofolliculomas are often diagnosed in guinea pigs, while other follicle-origin tumors, including trichoepithelioma, trichoblastoma, tricholemmoma, and pilomatricoma, can also be found [22,25,26,27]. In contrast to those in hamsters, hair follicle-originated tumors are not associated with viral infection. There is one case of thyroid adenocarcinoma in the present study. Both thyroid adenoma and adenocarcinoma are commonly observed in guinea pigs, and one of seven cases of malignant tumors metastasized to the lungs [26,28]. The role of thyroid disease in chronic degenerative disease in guinea pigs is unknown [28]. There are two cases of collagenous hamartoma in the present study. According to Dobromylskyj et al., the common non-inflammatory, non-neoplastic masses are follicular and epidermoid cysts, aural polyps, collagenous hamartomas, and salivary gland steatosis [26].

It is noteworthy that three of the four individuals with adenocarcinoma of the mammary gland of guinea pigs were males (Table 3). In contrast to those in other species, the prevalence of mammary gland tumors in both sexes of guinea pig was equal [29]. According to Dobromylskyj et al., 14 cases of malignant mammary tumors were diagnosed among 23 neutered male guinea pigs [26]. In one study, two cases of adenocarcinoma of the mammary gland were found in 19 pet guinea pigs, and all the individuals were male [27]. Together, these findings imply that male guinea pigs often develop malignant mammary gland tumors. Suárez-Bonnet et al. has found that the mammary tumors of guinea pigs were of a ductal origin, and the tumors were positive for estrogen and progesterone receptors, indicating that steroid hormones participate in oncogenesis [30]. In the present study, one case of uterine adenocarcinoma and one case of uterine lipoma were diagnosed. According to Greenacre [25], the most common tumor in the reproductive system is uterine leiomyoma, which is usually associated with cystic rette ovarii [25]. Nevertheless, the most common tumor found in another study including 83 guinea pigs with tumors and tumor-like lesions of the cervix and uterus is uterine adenoma, followed by leiomyoma and leiomyosarcoma [31].

The two rats in the present study had mammary gland adenocarcinoma and fibroadenoma of the mammary gland. The most common skin tumor in rats is fibroadenoma of the mammary gland, and adenocarcinoma is also common in rats [1]. The one 20-month-old gerbil in this study was diagnosed with cutaneous melanoma. In gerbils, the most common cutaneous tumors are SCC and melanomas [22]. It has been shown that gerbils commonly develop neoplasia after 24–36 months [1]. In a study involving 158 tumors from laboratory gerbils, the most common tumors in animals older than 36 months are sebaceous gland carcinoma and ovarian granulosa cell tumors [32].

A limitation of this collection is that it solely included the biopsy samples observed by the owners and clinicians, indicating an uncomprehensive collection. The diagnosis in this study relies mainly on histological features. Immunohistochemistry (IHC) to identify specific cell types has not been applied. One limitation of this study is the small case load, especially for MMTV detection. Although MMTV was undetectable in this study, it neither ruled out the role of MMTV in the development of rodent mammary tumors, nor the role of pet rodents in the cross-species MMTV transmission for the development of human mammary tumors. Although the positive control was valid (Figure 4), there are still technical details involving the testing of FFPE specimens. The major disadvantage of FFPE samples is that nucleic acids are easily degraded due to formalin fixation, leading to small DNA fragments. This result impairs the performance of RT-PCR. On the other hand, the positive control we used is cultured cells without formalin-fixed and paraffin-embedded processes. From the standpoint of clinicians, another limitation of this study is the lack of follow-up; therefore, the prevalence of metastasis or the prognosis of these malignant cases is unknown.

## 5. Conclusions

This study retrospectively collected 77 tumors and tumor-like lesions from 70 pet rodents in Taiwan. In 47 hamsters, the most common tumors included mammary tumors, fibrosarcoma, mast cell tumors, and squamous cell carcinoma. In 16 guinea pigs, the most common tumors included lipoma, adenocarcinoma of the mammary gland, and trichoblastoma. These findings have both similarities with and differences from those found in the published literature. We also, for the first time, tested all 20 rodent mammary tumors negative for MMTV. This study reveals that the common tumors in pet rodents are mammary tumors and the role of the MMTV infection in rodent mammary oncogenesis, as well as the role of pet rodents in the cross-species transmission of MMTV to humans, warrant further investigation.

## Figures and Tables

**Table 1:** Primer sets used for nested PCR detection of mouse mammary tumor virus nucleotide sequences.

Name	Primer Sequence (5′->3′)	Location	Expected PCR Product Size (bp)	No. of Cycles
ENV-1F	CCTTCTGGGAGGGAGACGAGT	*env*; 7273–7293; outer	647	35
ENV-1R	AGCTCGAATTAAATCTGTGGCATAC	*env*; 7895–7919; outer		
ENV-2F	CCTTGGGTTACTTTGGGATTTCTC	*env*; 7361–7384; inner	449	35
ENV-2R	TGATCGCTGCATAGTCGTAGGC	*env*; 7788–7809; inner		

**Table 2:** Neoplasms in pet hamsters of the current study.

			Sex	Age (Months)		
	Histologic Diagnosis	n	Male	Female	Range	Median
Integumentary	Malignant mammary tumor	10	0	10	3–24	11
	Fibrosarcoma	9	4	5	12–27	20
	Squamous cell carcinoma	4	4	0	7–18	19
	Malignant mast cell tumor	4	1	3	17–36	24
	Benign papilloma	3	3	0	15–23	18
	Mammary gland adenoma	3	0	3	14	14
	Malignant histiocytoma	2	1	1	12–24	18
	Myxosarcoma	1	1	0	19	19
	Pilomatricoma	1	1	0	17	17
	Sweat gland adenocarcinoma	1	1	0	24	24
	Trichoepithelioma	1	1	0	12	12
	Hemangiosarcoma	1	0	1	18	18
	Round cell tumor	1	0	1	na*	na
Reproductive	Luteoma	1	0	1	8	8
	Interstitial cell tumor	1	0	1	17	17
	Seminoma	1	1	0	18	18
	Uterine leiomyosarcoma	1	0	1	na	na
Urinary	Renal cell carcinoma	3	3	0	23–27	25
Hematopoietic	Splenic lymphoma	1	0	1	na	na
	Splenic fibrosarcoma	1	0	1	na	na

na*, not applicable.

**Table 3:** Neoplasms in pet guinea pigs of the current study.

			Sex		Age (Months)	
	Histologic Diagnosis	n	Male	Female	Range	Median
Integumentary	Lipoma	5	4	1	13–65	32
	Malignant mammary tumor	4	3	1	48–64	56
	Trichofolliculoma	2	0	2	28–60	44
	Sarcoma	1	1	0	68	68
	Collagenous hamartoma	2	2	0	68	68
	Sebaceous gland adenoma	1	1	0	55	55
Skeletal	Malignant giant cell tumor of bone	1	1	0	82	82
	Osteosarcoma	1	0	1	24	24
Reproductive	Uterine adenocarcinoma	1	0	1	17	17
	Lipoma	1	0	1	60	60
Endocrine	Thyroid adenocarcinoma	1	0	1	78	78

## Data Availability

The data presented in this study are available on request from the corresponding author. The data are not publicly available due to ethical restrictions.
